# Distortion Effect on the UHPC Box Girder with Vertical Webs: Theoretical Analysis and Case Study

**DOI:** 10.3390/ma17061303

**Published:** 2024-03-12

**Authors:** Chenguang Wang, Yaowen Wu, Yuanhai Zhang, Shiying Tang, Weiwen Li, Peng Wang, Walid Mansour

**Affiliations:** 1 School of Civil Engineering, Lanzhou Jiaotong University, Lanzhou 730070, China; wcgcivil@mail.lzjtu.cn (C.W.); zyh17012@163.com (Y.Z.); 2 Guangdong Provincial Key Laboratory of Durability for Marine Civil Engineering, Shenzhen University, Shenzhen 518060, China; 15602816784@163.com (Y.W.); tangshiying2018@email.szu.edu.cn (S.T.); waled_mansour@eng.kfs.edu.eg (W.M.); 3 Civil Engineering Department, Faculty of Engineering, Kafrelsheikh University, Kafrelsheikh 6860404, Egypt

**Keywords:** ultra-high performance concrete (UHPC), box girder, restrained torsion, distortion effect, parameter analysis

## Abstract

Distortion deformation usually imposes a potential threat to bridge safety. In order to comprehensively understand the distortion effect on thin-walled ultra-high performance concrete (UHPC) box girders, an innovative approach encompassing the governing distortion differential equation is introduced in this study based on the general definition of distortion angle within the cross-section plane. The analytical results obtained from the proposed method are in accordance with those obtained from the energy method, and exhibit favorable agreement with experimental findings documented in the existing literature. Furthermore, a finite element model is developed on the ANSYS 2021 R1 software platform with the employment of a Shell 63 element. Numerical outcomes are also in good agreement with the experimental data, affirming the validity and reliability of the findings. In addition, parameter analysis results indicate that the distortion angle remains approximately constant at a location approximately 1/10 of the span from the mid-span cross-section of the box girder, regardless of changes in the span-to-depth ratio. Increasing the web thickness yields a notable reduction in the distortion effects, and decreasing the wall thickness can effectively mitigate the distortion-induced transverse bending moment. Compared with normal-strength concrete box girders, UHPC box girders can reduce the distortion angle within the span range, which is beneficial for maintaining the overall stability of the box girders. The outcomes obtained from this study yield engineers an enhanced understanding of distortion effect on the UHPC girder performance.

## 1. Introduction

Single-cell box girders have become widely utilized in the construction of medium- and long-span highway bridges [1,2,3,4,5,6,7,8,9] due to their aesthetical appearance and exceptional resistance to bending and torsional forces [10,11,12,13,14,15,16,17]. The introduction of ultra-high performance concrete (UHPC), an advanced cementitious composite characterized by its low permeability and ultra-high strength [18], holds promising prospects for the transition of box girder towards thin-walled structures with larger spans and wider rib spacings [19,20,21,22,23,24]. Li et al. [25] showcased the UHPC girder bridge for the Fourth Beijiang River Bridge in Yingde City (in China), which featured a larger span (102 m) and reduced cross-section dimensions (15–20 cm). However, it was observed that the box girder exhibited notable warping effects. The cross-section of thin-walled box girder is susceptible to distortion under torsional effects, which may result in warping stress with a comparable magnitude to the longitudinal bending stresses. As a result, structural safety and passing persons are under the potential threat of distortion-induced damage. In light of this, it is imperative to uncover the distortion effect and address this issue with utmost seriousness.

In recent years, widespread attention has been devoted to investigating the distortional responses of thin-walled girders, with a particular focus on their mechanical properties and design methodologies. The integration of finite element techniques with thin-walled girder theory has led to the development of refined one-dimensional girder elements that incorporate distortion-related characteristics of the cross-section. These advancements have garnered considerable interest in the research community. Li et al. [26] introduced a novel one-dimensional refined girder finite element model that accounted for non-uniform distortional warping and the secondary deformations resulting from distortional moments. This model enabled the accurate analysis of distortional behavior in thin-walled box girders with cantilevered flanges. Zhao et al. [27] proposed a beam element with two torsional degrees of freedom (DOFs) and two distortional DOFs, specifically designed to investigate the eccentric load effects in box girder bridges. Zhu et al. [28] developed a one-dimensional model for a curved composite box girder and formulated a highly efficient finite girder element with 26 DOFs, which considered both constrained torsion and distortion. This element was particularly suitable for analyzing curved composite box girder. Additionally, Arici et al. [29] presented a practical analysis method that addressed the challenges associated with numerical approaches based on the finite element method. Their approach utilized the symplectic approach to overcome these difficulties effectively.

In addition to the above-mentioned finite element method, the theorem of the total potential energy variational method is commonly employed to derive the governing equilibrium equations in the analysis of distortional effects. Xu et al. [30] proposed a novel unified theory for distortion analysis of thin-walled hollow sections based on the Hellinger–Reissner energy variational principle. The results indicate that (1) the distortional shear deformation effects can be neglected in conventional hollow sections of bridge structures, and (2) the first derivative of the distortion angle can serve as an appropriate distortional warping function. It is worth mentioning that these two findings are the foundation of the proposed method in this study. Kermani et al. [31] have developed an elastic analysis method based on the stiffness approach to calculate the distortion effect, and the energy methods have been utilized to formulate the differential equation. Zhang et al. [32] derived analytical solutions for the distortion effects in the elastic stage of single-box structures, employing the energy variational principle. Campo et al. [33] established the governing differential equation using the principle of virtual works and presented a procedure to calculate the distortion effect in horizontally curved trapezoidal box girder bridges. While the aforementioned literature primarily focuses on warping normal stresses, it is important to also consider the distortion warping shear stresses to ensure the safety and serviceability of a thin-walled box girder. Moreover, the inclusion of distortion warping shear stresses provides a new perspective for analyzing the distortion effects of box girders. Stefanou et al. [34] determined the distortion shear flow by integrating the equilibrium equation. The distortion differential equation was developed comprehensively considering the distortion angle that arises from both distortion warping shear stress and external distortion loads. Building upon this methodology, Yoo et al. [35] developed an efficient procedure to evaluate the stresses resulting from distortion in horizontally curved box girders. 

While there is extensive research on analyzing the distortion effects of normal-strength concrete box girders, the UHPC girder has not been fully understood. In addition, there is relatively limited research on establishing distortion control differential equations using equilibrium conditions, leaving a challenging issue to theoretically uncover the underlying mechanisms within the cross-section of a distorted UHPC box girder. To address this issue, the main objective of this study is to develop an analytical method based on the equilibrium conditions to uncover the distortion effect on the UHPC box girder. This study defined the distortion angle of UHPC thin-walled box girders through the distortion center, and derived the formulas for the distortion normal stress and the distortion shear stress. Then, the distortion control differential equations were established through the total potential energy variational method and the deformation coordination method. The correctness of proposed deformation coordination method was validated by comparing its equations with that of total potential energy variational method. Finally, the distortion effect on UHPC thin-walled box girders was comprehensively studied by taking a case study as example. These findings yielded engineers with an innovative approach for analyzing the distortion effect on UHPC box girders, offering a comprehensive understanding and reasonable guidelines for practical engineering in this domain.

## 2. Distortion Warping Stresses

**Figure 1:**
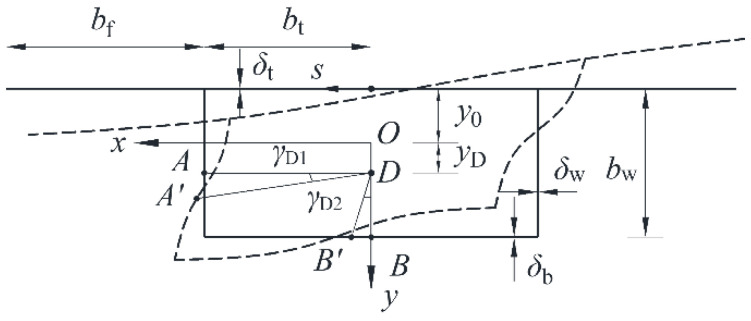
Distortion deformation of UHPC box girder.

The distortion deformation of a UHPC box girder is shown in Figure 1. Specifically, *b_t_* is the half length of top and bottom plates, *b_w_* is the girder height; *b_f_* is the width of flange plate, *δ_t_*, *δ_b_* and *δ_w_* are the thicknesses of top, bottom and web plates, respectively. *O_xyz_* is the centroidal principal coordinate system, and s is the circumferential coordinate, with the counterclockwise direction being positive. The distortion center is denoted as D, and points A and B correspond to the intersections of the horizontal and vertical lines, originating from point D, with the web and bottom plates, respectively. After the box girder is distorted, A and B will move to *A’* and *B’* and generate distortion angles of *γ*_D1_ and *γ*_D2_, respectively. The variation in ∠*ADB* (i.e., distortion angle *γ*_D_) is the sum of *γ*_D1_ and *γ*_D2_. 

Based on the distortion theory for thin-walled box beams, the analysis of distortion in UHPC box beams was conducted under the following fundamental assumptions [36,37,38,39,40,41,42]:

(1) The UHPC box beam was assumed to be in a linear elastic regime, neglecting the micro-cracks in the concrete.
(2) The normal and shear stresses of distortion warping were assumed to be unevenly distributed along the wall thickness.
(3) The UHPC box beam possessed a uniform cross-sectional configuration.
(4) The impact of prestress was disregarded in the analysis.

The tangential displacement (*u_t_*) of each plate in the s direction can be achieved by the distortion angle *γ*_D1_ and *γ*_D2_:

(1) $$u_{t}=\left\{\begin{array}{c} -\left(y_{0}+y_{D}\right)\gamma_{D2}\;\;\left(top\;plate\right)\\ -\left(b_{w}-y_{0}-y_{D}\right)\gamma_{D2}\;\;\left(bottom\;plate\right)\\ b_{t}\gamma_{D1}\;\;\;\;\left(web\;plate\right) \end{array}\right.$$

As the distortional shear deformation effects can be neglected, the shear strain $\gamma_{\mathrm{zs}}$ was roughly equal to zero. According to the geometric relationship, $\gamma_{\mathrm{zs}}$ can be expressed as

(2) $$\gamma_{\mathrm{zs}}=\frac{\partial u_{t}}{\partial z}+\frac{\partial w}{\partial s}=0$$

where w is the distortion warping displacement.

Substitute Equation (1) into Equation (2), the values of distortion angle $\gamma_{D1}$ and $\gamma_{D2}$ can be obtained based on the distortion warping displacement continuity condition ($\oint \frac{\partial u_{t}}{\partial z}ds=0$):

(3) $$\gamma_{D1}=\gamma_{D2}=\frac{\gamma_{D}}{2}$$

It can be seen from Equation (3) that for a vertical web box girder, the distortion angles of the two parts as defined in Figure 1 are the same.

Substituting Equation (3) into Equation (1), and assuming that the shear deformation is zero, it was observed that

(4) $$\frac{\partial w}{\partial s}=-\rho \gamma_{D}^{\prime }$$

where ρ was calculated as

(5) $$\rho =\left\{\begin{array}{c} -\left(y_{0}+y_{D}\right)/2\;\;(top\;plate)\\ -\left(b_{w}-y_{0}-y_{D}\right)/2\;\;(bottom\;plate)\\ b_{t}\;\;\;\;\;(web\;plate) \end{array}\right.$$

We integrated the perimeter coordinates s on both sides of Equation (5),

(6) $$w=-\gamma_{D}^{\prime }\omega_{D}$$

where $\omega_{D}=\int_{0}^{s}\rho ds$ is the distortional warping sectorial coordinate. According to the geometric equation, the distortion warping stress ($\sigma_{D}$) on the cross-section of box girder can be expressed as

(7) $$\sigma_{D}=-E\gamma_{D}^{\prime \prime }\omega_{D}$$

According to the self-balance condition that the distortion warping normal stress does not synthesize axial force and bending moment on the cross-section of box girder, the ratio β of the distortional warping sectorial coordinates can be computed for the intersection points of web plate with the top and bottom plates:

(8) $$\beta =\frac{A_{b}+3A_{w}}{A_{t}{\left(1+\frac{b_{f}}{b}\right)}^{2}+3A_{w}}$$

where $A_{t}=2(b+b_{f})\delta_{t}$ is the area of top plate area, $A_{b}=2b\delta_{b}$ is the area of bottom plate, $A_{w}=bw\delta_{t}$ is the area of web plate.

According to the definition of the distortional warping sectorial coordinate, the centroid coordinate position *y*_0_ and distortion center position coordinate $y_{D}$ meet the following relationship:

(9) $$\beta =\frac{y_{0}+y_{D}}{b_{w}-\left(y_{0}+y_{D}\right)}$$

**Figure 2:**
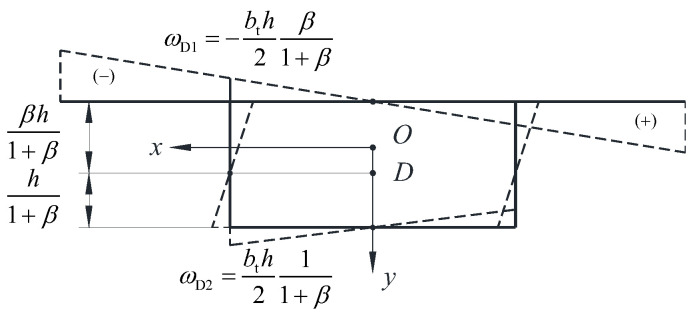
Distortional warping sectorial coordinate distribution.

It is worth noting that, β equal to the ratio of the upper and lower parts of the girder height divided by the distortion center *D*, as shown in Figure 2. For the vertical web box girder, when the plates are bent out of plane, the points *A* and *B* are the reverse bending points, i.e., the zero point of the distortional warping sectorial coordinate. 

**Figure 3:**
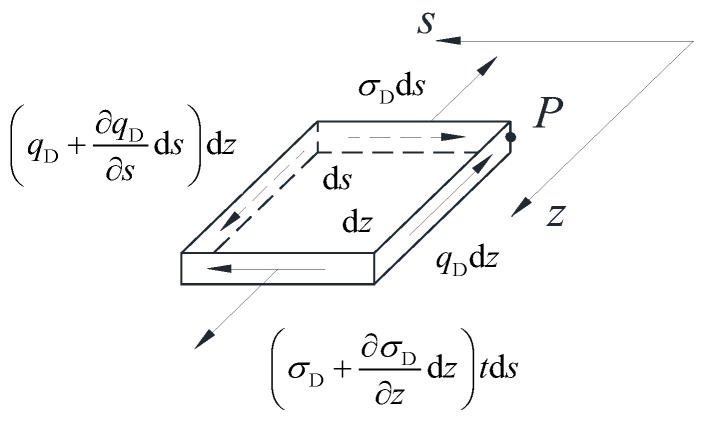
Thin-walled box girder box wall microelement force diagram.

The micro element of dz×ds was selectively extracted along the longitudinal and circumferential orientations of the thin-walled box girder, as depicted in Figure 3. Based on the equilibrium condition of the micro element on the box wall, a relationship can be established between the distorted warping normal stress $\sigma_{D}$ and the distorted warping shear flow $q_{D}$:

(10) $$\frac{\partial q_{D}}{\partial s}=-\frac{\partial \sigma_{D}}{\partial z}t$$

We integrated Equation (10), and the distortion warping shear flow $q_{D}$ could be obtained as

(11) $$q_{D}=q_{D0}+E\gamma_{D}^{\prime \prime \prime }S_{\mathrm{WD}}$$

where $q_{D0}$ is constant shear flow, $S_{\mathrm{WD}}=\int_{0}^{s}\omega_{D}tds$ is the distorted fan static moment, and *E* is the modulus of elasticity. The distortion warping shear flow $q_{D}$ does not form torque on the cross section of box girder, i.e., $\int_{A}q_{D}\rho ds=0$

Given this, the constant shear flow $q_{D0}$ can be obtained as

(12) $$q_{D0}=EC\gamma_{D}^{\prime \prime \prime }$$

where

(13) $$\begin{array}{c} C=-\frac{1}{4b_{t}b_{w}}\int_{A}S_{\mathrm{WD}}\rho ds\\ \;\;\;\;\;\;\;\;\;\;\;=-\frac{\omega_{D1}}{4}\left[\left(1+\frac{b_{f}}{b_{t}}\right)A_{t}+\frac{A_{w}}{3}\left(5-4\frac{1}{\beta }\right)-\frac{1}{3\beta }A_{d}\right] \end{array}$$

Substituting Equation (12) into Equation (11), the distortion warping shear flow $q_{D}$ can be expressed as

(14) $$q_{D}=CE\gamma_{D}^{\prime \prime \prime }+S_{\mathrm{WD}}E\gamma_{D}^{\prime \prime \prime }=q_{D0}+q_{\mathrm{DW}}$$

The distortion warping shear flow $q_{D}$ will form distortion moment $M_{D}$ within the cross section:

(15) $$M_{D}=2b_{t}\int_{b_{t}}^{b_{t}+b_{w}}q_{D}ds=WE{\gamma_{D}}^{\prime \prime \prime }$$

(16) $$W=\frac{b_{t}^{2}b_{w}^{2}}{12\left(1+\beta \right)}\left[\left(2-\beta \right)A_{w}+A_{b}\right]$$

where *W* is the geometric parameter representing the distortion resistance of box girder.

It can be seen from Equation (14) that the distribution of distortion warping shear flow $q_{D}$ within the cross section plane is only dependent on the distortional sectorial static moment $S_{\mathrm{WD}}$. The distorted fan static moment $S_{\mathrm{WDW}}$ on the left web can be expressed as

(17) $$\begin{array}{c} \;\;\;S_{\mathrm{WDW}}=S_{\mathrm{WD}1}+\int_{0}^{s}\left(\omega_{D1}+\frac{\omega_{D2}-\omega_{D1}}{h}\right)\delta_{w}ds\\ =S_{\mathrm{WD}1}+\left(s-\frac{1+\beta }{2\beta h}s^{2}\right)\delta_{w} \end{array}$$

where $S_{\mathrm{WD}1}$ is the distorted fan static moment at the top of the web.

When the first-order derivative of Equation (17) equals zero, the distortion warping shear flow on the web will achieve an extreme value.

(18) $$s=\frac{\beta h}{1+\beta }$$

Equation (18) shows that for the vertical web thin-walled box girder, the intersection a and B of the horizontal line and vertical line passing through the distortion center D point and the web and bottom plate of the box girder are not only the zero point of the distortion warping normal stress, but also the extreme point of the distortion warping shear stress.

## 3. Total Potential Energy Variational Method

The total potential energy of distortion consists of three parts: distortion warping strain energy, distortion frame strain energy, and external load potential energy [43,44,45,46,47,48,49,50,51]. This derivation follows the principle of minimum potential energy. Based on Equation (7), the distortion frame strain energy *U*_1_ can be computed as

(19) $$U_{\sigma }=\frac{1}{2E}\int_{0}^{l}\int_{A}\sigma_{D}^{2}dAdz=\frac{EI_{\mathrm{DW}}}{2}{\int_{0}^{l}\left(\gamma_{D}^{\prime \prime }\right)}^{2}dz$$

where $I_{\mathrm{DW}}=\int_{A}\omega_{D}^{2}dA$ represents the distortion-resistant bending moment, which can be expressed as

(20) $$I_{\mathrm{DW}}=\frac{b_{t}^{2}b_{w}^{2}}{12\left(1+\beta \right)}\left[\left(2-\beta \right)A_{w}+A_{b}\right]$$

Through a comparison of Equations (16) and (18), *I*_DW_ can be further expressed as $I_{\mathrm{DW}}=W$.

$K_{D}$ represents the stiffness of the distortion-resistant frame, i.e., the distortion moment $M_{\gamma D}$ required to generate a unit distortion angle within the thin-walled box girder:

(21) $$M_{\gamma D}=K_{D}\gamma_{D}$$

where $K_{d}=\frac{24EI_{1}}{h\zeta_{0}}$, $\zeta_{0}=1+\frac{2\frac{b}{h}+3\frac{I_{2}+I_{4}}{I_{1}}}{\frac{I_{2}+I_{4}}{I_{1}}+6\frac{hI_{2}I_{4}}{bI_{1}^{2}}}$,

$I_{1}$, $I_{2}$ and $I_{3}$ represent the out-plane moment of inertia of the unit length frame for top, bottom and web rods, respectively.

Thus, the distortion frame strain energy $U_{\tau }$ can be determined as

(22) $$U_{\tau }=\frac{1}{2}\int_{0}^{l}M_{\gamma D}\gamma_{D}dz=\frac{K_{D}}{2}\int_{0}^{l}\gamma_{D}^{2}dz$$

Eccentricity load P with eccentricity e generate $m_{t}=Pe$ and $M_{\mathrm{DV}}=\frac{m_{t}}{2}$ within the cross-section of box girders with internal diaphragms.

In light of this, the distortion-induced external load potential energy $V_{m}$ can be expressed as

(23) $$V_{m}=-\int_{0}^{l}M_{\mathrm{DV}}\gamma_{D}dz=-\int_{0}^{l}\frac{m_{t}}{2}\gamma_{D}dz$$

The total potential energy of distortion of box girders with internal diaphragms $V_{m}$ can be expressed as

(24) $$\Pi =U_{\sigma }+U_{\tau }+V_{m}$$

Substituting Equations (17), (20) and (21) into Equation (22), the governing distortion differential equation can be derived due to the fact that the first-order variation of total potential energy (δΠ) is equal to zero.

(25) $$EI_{\mathrm{DW}}{\gamma_{D}}^{\prime \prime \prime \prime }+K_{D}\gamma_{D}=\frac{m_{t}}{2}$$

## 4. Deformation Coordination Method

The distortion-induced warping normal stresses, distortion-induced warping shear stresses, and externally induced distortion moment can generate distortion angles separately. When using the deformation coordination method to analyze the distortion effect of thin-walled box girders, a unit length thin-walled frame was taken along the girder span direction. Therefore, the distortion moment acting on the thin frame should be its variation rate along the longitudinal direction of the UHPC girder. According to Equation (15), the distortion angle $\gamma_{\tau }$ generated by the distortion warping shear flow is

(26) $$\gamma_{\tau }=\frac{WE{\gamma_{D}}^{\prime \prime \prime \prime }}{K_{D}}$$

Similarly, the distortion angle $\gamma_{m}$ caused by distortion external load is

(27) $$\gamma_{m}=\frac{m_{t}}{2K_{D}}$$

Based on the deformation coordination condition of distortion angles, the distortion angle generated by the UHPC box girder under external distortion loads is equal to the distortion angle jointly produced by distortion bending normal stress and distortion bending shear stress. The distortion angle deformation coordination equation was established as follows:

(28) $$\gamma_{D}=\gamma_{\tau }+\gamma_{m}$$

Substituting Equations (24) and (25) into Equation (26), the governing distortion differential equation for UHPC box girder was obtained based on the deformation coordination method:

(29) $$WE{\gamma_{D}}^{\prime \prime \prime \prime }+K_{D}\gamma_{D}=\frac{m_{t}}{2}$$

As $I_{\mathrm{DW}}=W$, it is evident that Equations (25) and (29) are equivalent.

## 5. Numerical Examples

**Figure 4:**
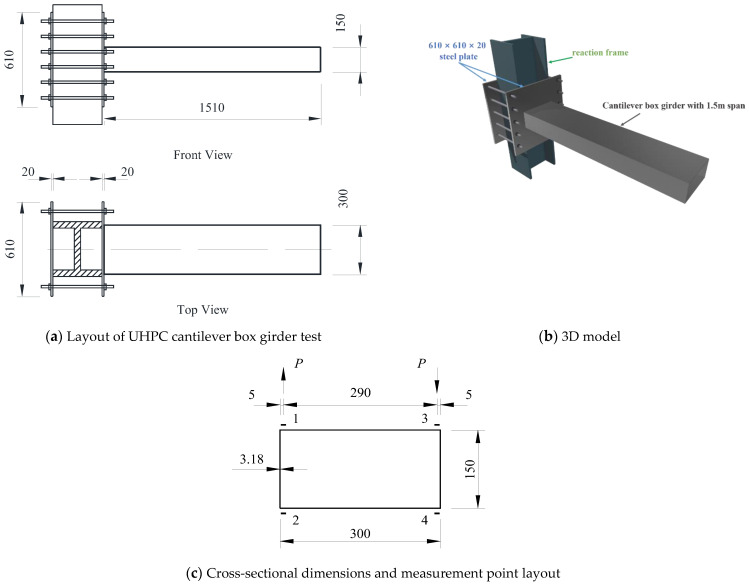
Configuration of cantilever box girder (unit: mm).

In the previous literature [36], a bench scale test of a cantilever box girder was conducted to investigate the distortion effects. The specific configuration of a tested cantilever box girder is shown in Figure 4. The cross-section of the girder was *b* × *h* = 300 mm × 150 mm, with a wall thickness of 3.18 mm. The employed cold-rolled low-carbon steel plate with dimensions of 610 × 610 × 20 mm^3^ had an elastic modulus of *E* = 196.2 GPa. The measurement section was chosen as 3/4 of the span from the free end, where the torsion-induced warping stress was minimal, making the measured warping stress values representative.

Furthermore, a finite element model was developed on the ANSYS 2021 R1 software platform using the Shell 63 element. As shown in Table 1, the numerical results of the cantilever box girder were in good agreement with the experimental and analytical results with acceptable errors (<10%), confirming the correctness of the proposed calculation methods in this paper.

**Figure 5:**
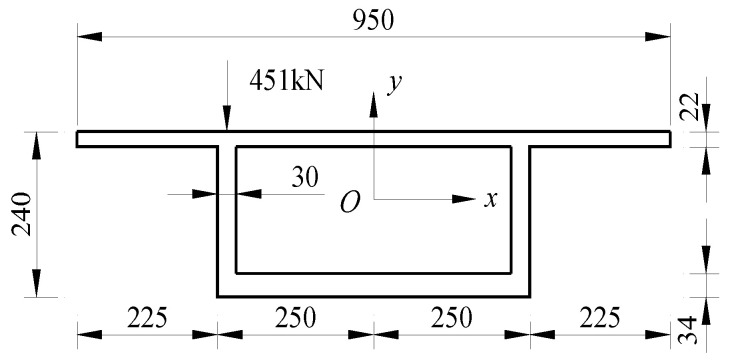
Simple UHPC support box girder (cm).

In addition, a parametric study was conducted by taking a simply supported box girder bridge as an example. As shown in Figure 5, the bridge span (*l*) was 40 m in length. The box girder was made of UC120 concrete, with an elastic modulus of *E* = 43 GPa. An eccentric load of P = 451.0 kN was applied at the top-left corner on the mid-span cross-section of the box girder.

**Figure 6:**
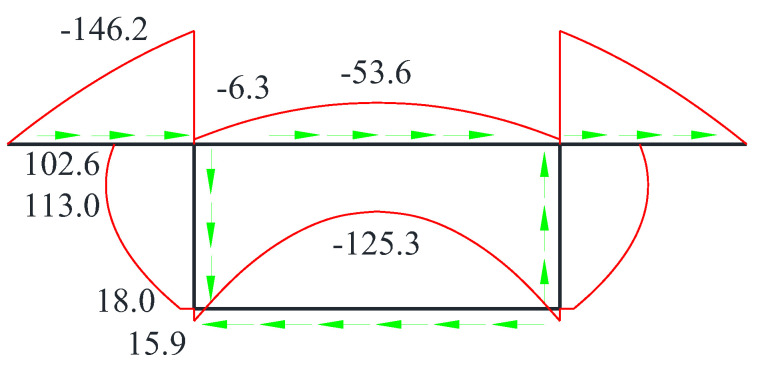
Distribution of distortion-induced warping shear stresses (green arrows denote the shear stress flow).

The distribution of distortion-induced warping shear stresses is illustrated in Figure 6. The magnitude of the distortional warping shear stress was symmetrically distributed within the cross-section. On both sides of the cantilever plate, the distortional warping shear stress was in the same direction. In the closed box, the distortional warping shear stress directions on the top and bottom plates and the left and right flanges were opposite. The peak value of the distortional warping shear stress was achieved at the mid-point of the top and bottom plates. The shear force on the cantilever plate reduced when approaching to the end of the cantilever plates. The peak shear stress on the flange was achieved at the mid-point of web plates. In light of this, necessary strengthening measures can be taken at the mid-points of top, bottom and web plates to effectively reduce the distortional warping shear stress within the cross-section plane.

**Figure 7:**
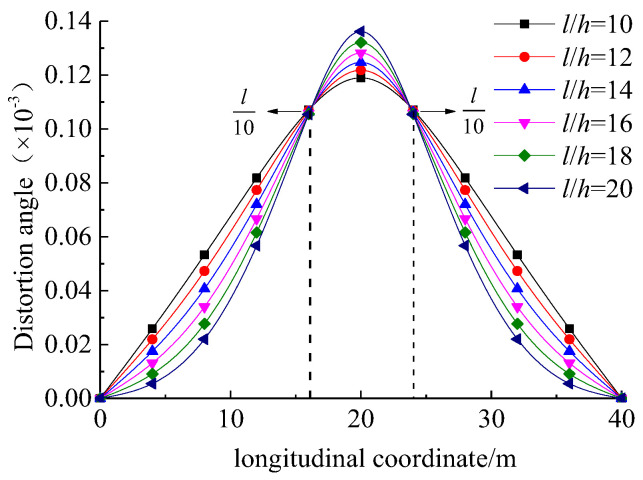
Variation in distortion angle with respect to the span-to-height ratio.

Figure 7 illustrates the variation in distortion angle within the cross-section of girder with respect to the span-to-height ratio (l/h). It can be observed that the maximum distortion angle in the box girder occurred at the mid-span, and gradually decreased towards both ends of UHPC box girder. Moreover, the maximum distortion angle increased with l/h. It is worth mentioning that there were stationary points at approximately l/10 from the mid-span of UHPC box girder.

**Figure 8:**
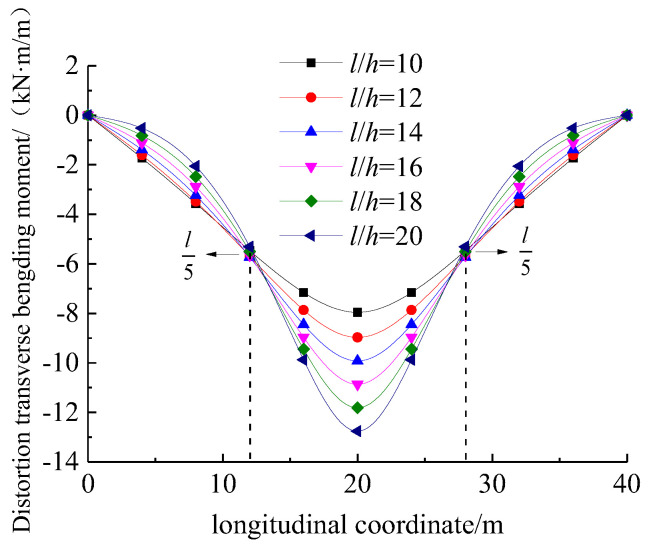
Variation in distortion-induced transverse bending moment with respect to the span-to-height ratio.

Figure 8 illustrates the variation in the distortion transverse bending moment at the loaded point with respect to the span-to-height ratio (l/h). It can be observed that it shows a similar trend to the distortion angle. It can be observed that the maximum distortion warping normal stress in the UHPC box girder occurred at the mid-span point, and its peak value significantly increased with l/h. It is worth mentioning that there were stationary points at approximately l/5 from the mid-span of UHPC box girder.

**Figure 9:**
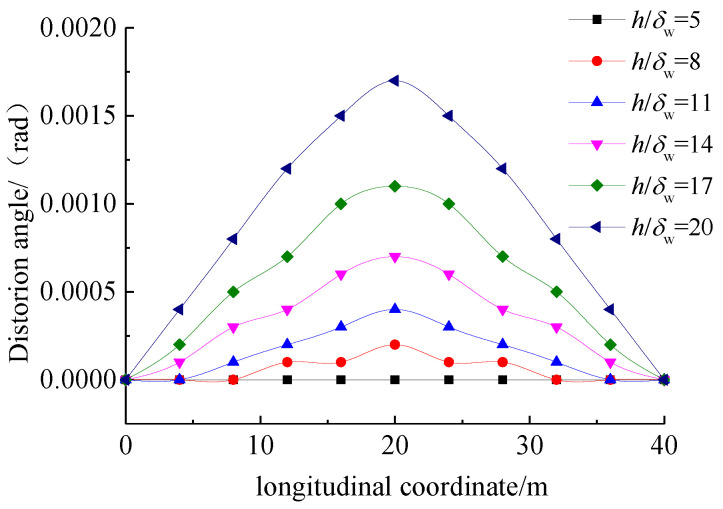
Variation in distortion angle with respect to the height-to-thickness ratio.

Figure 9 illustrates the variation in distortion angle at the cross-section of the girder with respect to the height-to-thickness ratio of the web pate ($h/\delta_{w}$). With an increase in $h/\delta_{w}$, the distortion angle significantly increased and gradually decreased towards both ends of the UHPC girder. Therefore, the increase in web thickness was an effective approach to reduce the distortion effects.

**Figure 10:**
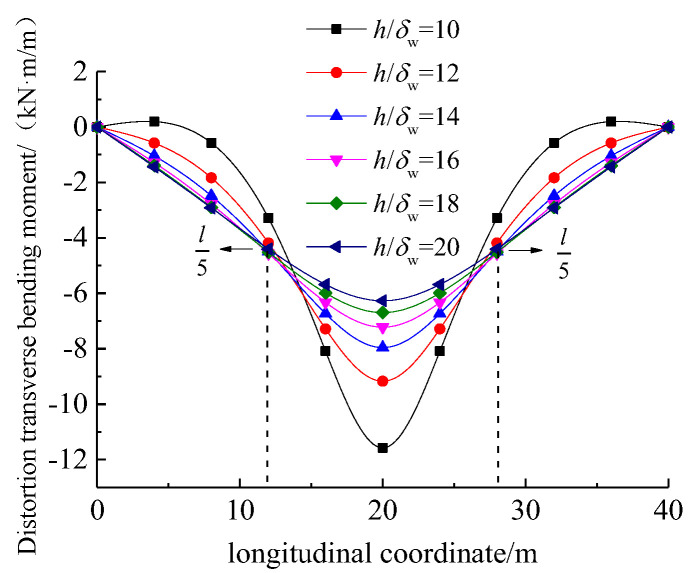
Variation in distortion transverse bending moment with respect to height-to-thickness ratio.

Figure 10 illustrates the variation in distortion lateral load at the loaded point with the height-to-thickness ratio of the web plate ($h/\delta_{w}$). It can be observed that the distortion-induced transverse bending moment gradually decreased with a larger $h/\delta_{w}$. It is worth mentioning that there were stationary points at approximately l/5 from the mid-span of UHPC box girder. Therefore, the reduction in web thickness $\delta_{w}$ was an effective approach to reduce the distortion-induced transverse bending moment.

**Figure 11:**
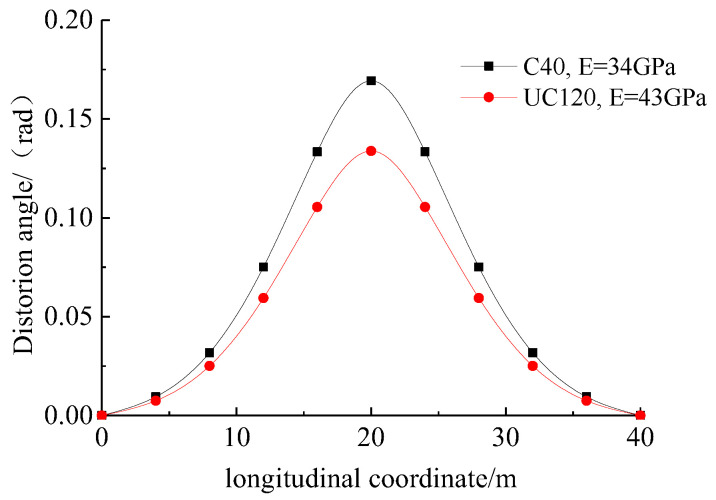
Comparison between normal-strength concrete and UHPC box girder in terms of distortion angle.

Figure 11 illustrates the variation in the distortion angle within the span range of the normal-strength concrete and the UHPC box girders with the same dimensions (Figure 5). The normal-strength concrete exhibited a compressive strength of 40 MPa and an elastic modulus of 34 GPa. Compared to the normal-strength concrete counterpart, the UHPC box girders could effectively reduce the distortion angle and exhibit enhanced overall stability.

## 6. Conclusions

This paper introduced an analytical approach to analyze the distortion effect, utilizing the distortion angle of the UHPC box girder. Furthermore, a novel method was proposed based on the equilibrium conditions of deformation coordination. A governing differential equation was derived by employing energy variation calculus grounded in the principle of minimal potential energy. The proposed method was validated by comparing its results with those obtained through the energy method and experimental findings from the existing literature. Additionally, parametric studies were conducted to examine the impact of variations in geometric parameters on the distortion effects of thin-walled box girders. Through comprehensive analysis, several major conclusions were drawn as follows:

(1) For vertically web-plated UHPC box girders, the mid-points on the top, bottom and web plates showcased zero distortional warping normal stress and extreme distortional warping shear stress.
(2) The theoretical derivation process demonstrated that the distortional governing differential equations derived from both the total potential energy variational method and the deformation coordination method were entirely identical, confirming the correctness of the proposed deformation coordination method.
(3) At a distance approximately equal to 1/10 of the span from the middle position of the UHPC box girder, the distortion angle remained constant regardless of the span-to-depth ratio. The distortion effects of the UHPC box girder could be significantly mitigated by increasing the web thickness. Moreover, reducing the thickness of box walls could effectively alleviate the distortion-induced transverse bending moment.
(4) Compared with the normal-strength concrete box girders (C40), the UHPC box girders (UC120) could significantly reduce the distortion angle within the span range, which was beneficial for maintaining the overall stability of the box girders.

Although this study yielded fruitful outcomes, it is important to acknowledge certain limitations. The methodology and associated formulas presented in this study were specifically tailored for the analysis of distortion effects in single-cell box girders featuring vertical web plates. To expand the applicability of this method, future research endeavors could focus on extending the proposed approach to encompass the examination of distortion effects in multi-cell box girders incorporating inclined web plates. Furthermore, the analysis conducted in this study exclusively examined the distortion effects on UHPC box girders under simplified loading conditions. To gain a deeper understanding of the distortion effects under more complex loading conditions, future research should incorporate the nonlinear material properties of both steel and concrete. Additionally, while this paper focused solely on UHPC box girders, it is crucial to extend the investigation to encompass other types of girders, thereby verifying the broader applicability of the proposed model. Despite these limitations, this study provides valuable insights for engineers concerning the distortion effects on UHPC box girders.

## Figures and Tables

**Table 1:** Distortion warping normal stress (unit: MPa).

Measurement Point	Analytical Solution	Numerical Solution	Experimental Value	Δ_1_ (%)	Δ_2_ (%)
1	−33.26	−34.05	−31.62	4.93	7.14
2	33.26	34.05	32.08	3.55	5.79
3	33.26	34.05	32.55	2.14	4.41
4	−33.26	−34.05	−33.42	0.48	1.85

Note: Δ_1_ = (Warping stress analytical solution − Warping stress experimental value)/Warping stress analytical solution × 100%; Δ_2_ = (Warping stress finite element solution − Warping stress experimental value)/Warping stress finite element solution × 100%.

## Data Availability

The raw data required to reproduce these findings are available only with direct contact through email to the corresponding author. The processed data required to reproduce these findings are available only with direct contact through email to the corresponding author.
